# Self-organized phase-transition lithography for all-inorganic photonic textures

**DOI:** 10.1038/s41377-021-00534-5

**Published:** 2021-04-29

**Authors:** Bo Zhang, Dezhi Tan, Zhuo Wang, Xiaofeng Liu, Beibei Xu, Min Gu, Limin Tong, Jianrong Qiu

**Affiliations:** 1 State Key Laboratory of Modern Optical Instrumentation, College of Optical Science and Engineering, Zhejiang University, 310027 Hangzhou, China; 2 School of Materials Science and Engineering, Zhejiang University, 310027 Hangzhou, China; 3 Centre for Artificial-Intelligence Nanophotonics, School of Optical Science and Engineering, Shanghai University of Science and Technology, 200093 Shanghai, China; 4 CAS Center for Excellence in Ultra-intense Laser Science, Chinese Academy of Sciences, 201800 Shanghai, China

**Keywords:** Photonic crystals, Laser material processing, Lithography

## Abstract

Realizing general processing applicable to various materials by one basic tool has long been considered a distant dream. Fortunately, ultrafast laser–matter interaction has emerged as a highly universal platform with unprecedented optical phenomena and provided implementation paths for advanced manufacturing with novel functionalities. Here, we report the establishment of a three-dimensional (3D) focal-area interference field actively induced by a single ultrafast laser in transparent dielectrics. Relying on this, we demonstrate a radically new approach of self-organized phase-transition lithography (SOPTL) to achieve super-resolution construction of embedded all-inorganic photonic textures with extremely high efficiency. The generated textures exhibit a tunable photonic bandgap (PBG) in a wide range from ~1.3 to ~2 μm. More complicated interlaced textures with adjustable structural features can be fabricated within a few seconds, which is not attainable with any other conventional techniques. Evidence suggests that the SOPTL is extendable to more than one material system. This study augments light–matter interaction physics, offers a promising approach for constructing robust photonic devices, and opens up a new research direction in advanced lithography.

## Introduction

Interference phenomena, ubiquitous in light–light and light–matter interactions, allow for manipulation of the distributions of the electromagnetic field and manufacturing of functional structures^1–5^. Some of the well-known examples include multibeam interference^1,6^, holographic interference^7,8^, surface-plasmon polariton (SPP) interference^9,10^, and interference between the incident light and scattered waves from defects or inhomogeneities^11,12^, enabling patterning periodic structures with high efficiency and throughput. Even though various interference fields have been considered as well-established processing tools on the surface, the 3D spatial interference lithography inside inorganic transparent dielectrics, however, remains very difficult or even impossible without the use of complex beam-shaping manipulation or photosensitizers. Therefore, robust optical elements and embedded photonic integration are lacking. Ultrafast laser–matter interaction has been considered a treasure trove for a variety of novel physical phenomena that may give solutions to current “impossible tasks”^13–17^. Theoretically, an intense spatial interference field can be actively excited by single-beam ultrafast laser irradiation and may possess the ability of nonlinear material modification. This provides a potentially flexible and high-efficiency shortcut that can quickly generate 3D super-resolution interference lithography in transparent dielectrics without any additional setups or photosensitive components. However, as a pioneering scenario that has not been experimentally demonstrated, its principle and feasibility remain unclarified.

Photonic crystals (PCs), which play an immense role in photon-control engineering^18–21^, are constructed by periodically organized artificial textures with a feature size comparable to the light wavelength^22–24^. In particular, creating near-infrared light-responsive textures is highly valuable for a wide scope of research fields like optical communication, computing, and sensing^25–27^. However, despite more than 30 years of research and numerous publications on photonic textures by processing photoresists^28,29^, semiconductors^30,31^, and metals^32,33^, there is no effective method that has been established to massively produce all-inorganic photonic textures using a single-step process. This is one of the greatest limiting factors to the further development of PCs. Besides, achieving photonic textures having a tunable PBG inside transparent dielectrics remains a major challenge for current research and an important component that is missing in advanced nanostructuring.

We recently discovered an unprecedented 3D interference field actively excited by ultrafast laser–matter interaction in La_2_O_3_–Ta_2_O_5_–Nb_2_O_5_ glass (LTN glass) that allows the realization of super-resolution lithography of highly regular embedded crystalline photonic textures with tunable optical performances in the near-infrared region. Based on this, a series of photonic structures can be created quickly with a simple one-step process, and the method has been demonstrated to possess a considerable degree of universality in different transparent dielectrics, from glasses to crystals. The problem of high-efficiency construction of complicated all-inorganic 3D photonic elements in transparent dielectrics is resolved.

**Fig. 1:**
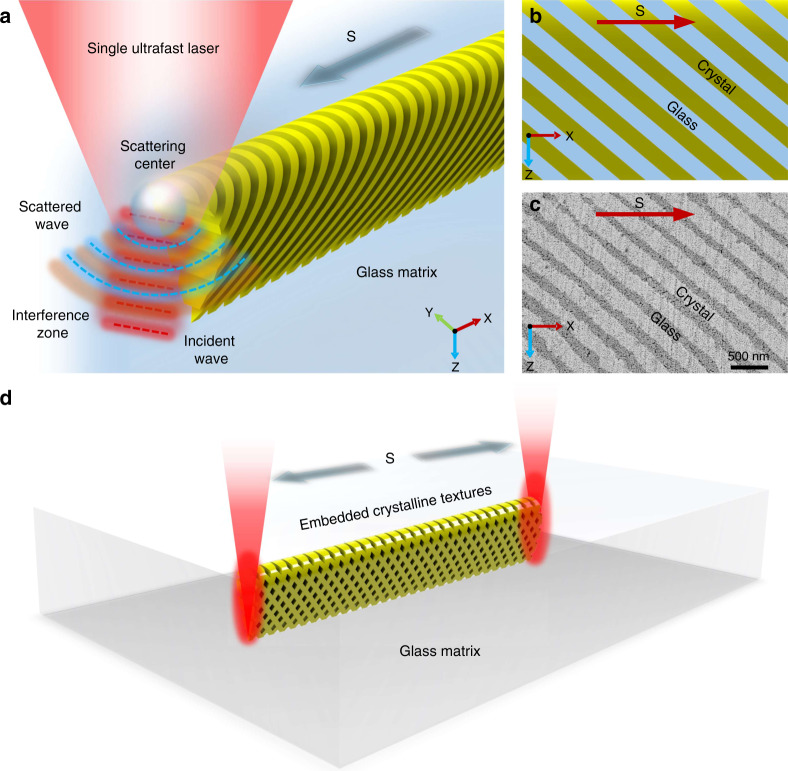
Conceptual model of the SOPTL fabrication. **a** Schematic diagram of the photonic texture formation by the interference field as the single-beam laser is scanning in the glass matrix. **b** Schematic diagram of the expected morphology of the created photonic textures. **c** High-resolution scanning electron microscopy (SEM) image of the created textures. **d** Schematic diagram of more complicated crystalline texture construction by the SOPTL. The arrows represent the beam scanning direction (denoted by S)

Here, we present a unique 3D interference phenomenon between the focused ultrafast laser and scattered spherical wave based on the light–matter interaction in the glass to give a precise description of the interference field distribution with a single scattering center model (Supplementary Fig. S2). By exploiting this principle, a strategy of ultrafast laser-induced SOPTL is established to efficiently create large-scale periodic photonic textures (Supplementary Movie S1). In our design, ultrafast laser focusing in transparent media, under proper conditions, can produce a scattering center in the focal area due to the strong multiphoton ionization caused by local modification^34,35^, making it possible to establish an intense scattered light field. The spatial interference in three-dimension between the incident and scattered wave occurs, creating a holographic light field (Fig. 1a). In the glass, this triggers periodic crystallization with the beam-writing process and enables regularly patterned crystal precipitation in 3D along the writing path (Fig. 1b, c). By manipulating the focal light field in the SOPTL, more complex photonic textures can be created with remarkable high efficiency (Fig. 1d). More importantly, an obvious PBG at communication band is produced, which relies on the large refractive index contrast between the crystal phase and the glass matrix, and the PBG is tunable in an extremely wide range (~700 nm) through the engineering of a secondary-phase transition. We believe that with the inception of the SOPTL concept, more efficient methods and functional materials aimed at large-scale production of all-inorganic photonic textures will be developed in the near future, and present a revolutionary approach to develop embedded photonic devices in transparent media.

## Results

**Fig. 2:**
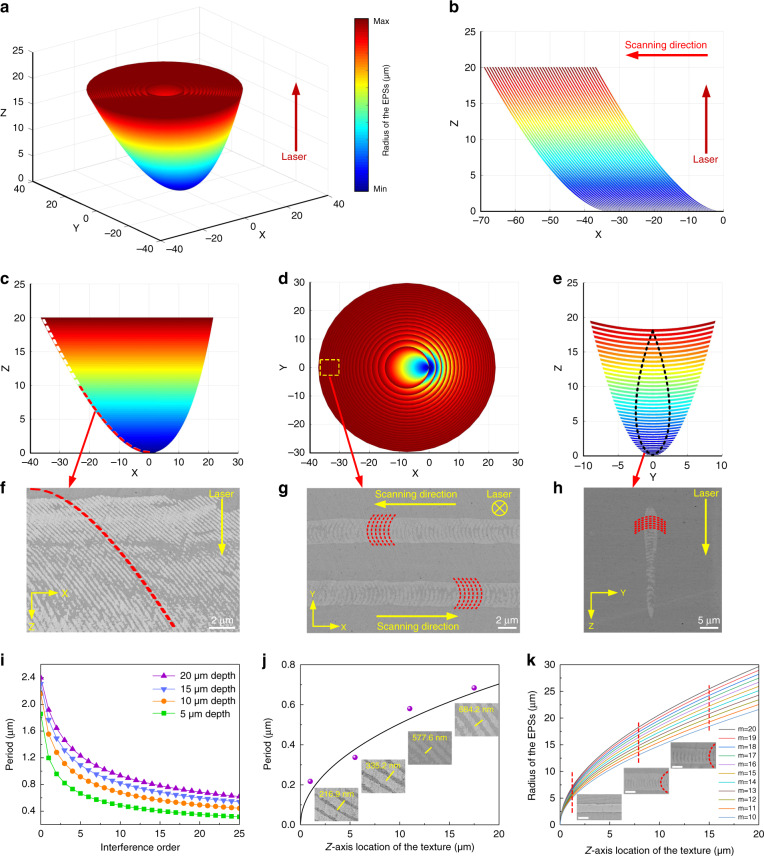
3D interference and mechanism of texture generation. **a** The calculated EPSs of the 3D interference field. **b** The simulated texture constructed by the SOPTL in the *XZ* plane. **c** The *XZ* view of the EPSs. The dotted curve is the calculated edge of an equal-phase face. **d**, **e** The *XY* view (**d**) and *YZ* view (**e**) of the EPSs. The dotted area shows the EPSs work in the SOPTL. **f** SEM image of the generated photonic textures in the *XZ* plane. The dotted curve is the calculated edge of the equal-phase face. **g**, **h** SEM images of the photonic textures in the *XY* plane (**g**) and *YZ* plane (**h**). The dotted curves help to visualize the curved stripes in the actual photonic textures corresponding to the simulated results. **i** Periods of the photonic textures at different depths as a function of the interference order. **j** Calculated period of the photonic textures as a function of depth with the interference order set as 20. Insets are the observed periods, corresponding to the purple points in the image. **k** Radii of the EPSs with different interference orders as a function of depth. Insets are the *XY* view of the photonic textures at different depths (indicated by the dotted lines). The dotted curves in the insets help to visualize the bending degree. Scale bar: 2 µm

Figure 2a demonstrates the theoretical spatial distribution of the light interference field represented as a series of equal-phase surfaces (EPSs) for constructive interference. When the ultrafast laser beam moves relative to the sample, specific EPSs participate in the SOPTL process due to the limitation of the crystallization condition, leading to the formation of periodic structures. Figure 2b shows the theoretical morphology of the periodic structures in the XZ plane created by the SOPTL (more details about the theoretical derivation and experimental evidence are found in the supplementary information). As expected, the experimentally observed periodic structures, revealed by the intersecting patterns in view planes (*XZ*, *XY*, and *YZ* plane), are consistent with the theoretical results, which confirms the validity of our proposal.

Figure 2c illustrates the curve extracted from the edge of the *XZ* projection of an equal-phase face, which corresponds to the pattern of the experimentally observed periodic arrays in Fig. 2f. Notably, the interference order of the equal-phase face derived here is as high as 20, which is the root cause of the inclination of the periodic structures in the XZ plane. Meanwhile, from the *XY* view of the generated textures, arcuate fringes that are similar to the calculated EPSs are observed (Fig. 2d, g). As expected, such arcuate fringes exhibit an obvious dependence on the laser scanning direction, strongly supporting the concept that only EPSs with certain interference orders along the specific orientation participate in the formation of the periodic structure. Furthermore, as a result of the periodic arrangement of EPSs along the beam-writing path, the *YZ* view of the textures is theoretically characterized by a series of arcuate fringes along the laser propagation direction (Fig. 2e), in line with experimental observation (Fig. 2h).

The theoretical dependence of the periods at different depths along the *Z* axis on the interference order is clarified in Fig. 2i, which indicates that the period of the texture varies with the interference order, and also increases with the depth along the laser propagation direction. Figure 2j shows the theoretical relationship between the period and depth with the interference order set as 20, coinciding with actual values (purple points), which further points to the effective EPSs responsible for the SOPTL process. Based on the theoretical model, the radius of the interference fringes gradually increases with depth, inevitably leading to a smaller bending degree of the periodic stripes in the *XY* plane, which is in line with experimental results (Fig. 2k). The simulation indicates that all the EPSs merge in the original focal plane at the depth of zero, meaning that the periodic feature will theoretically disappear. This prediction is verified by SEM observation at the top area of the periodic structures (Fig. 2k, insets). Furthermore, the inclination direction of the periodic structures is dependent on the laser scanning direction and always tilted to the starting point of laser scanning. Supplementary Fig. S3a shows a nearly perfect symmetry between the periodic textures written by scanning the ultrafast laser in opposite directions from the same starting point, which echoes the direction-dependent arcuate fringes observed in the *XY* view (Fig. 2g). Taken together, both qualitative and quantitative results confirm that a well-defined 3D light interference is established in the focal area and the SOPTL is triggered to effectively build complex spatial periodic textures.

**Fig. 3:**
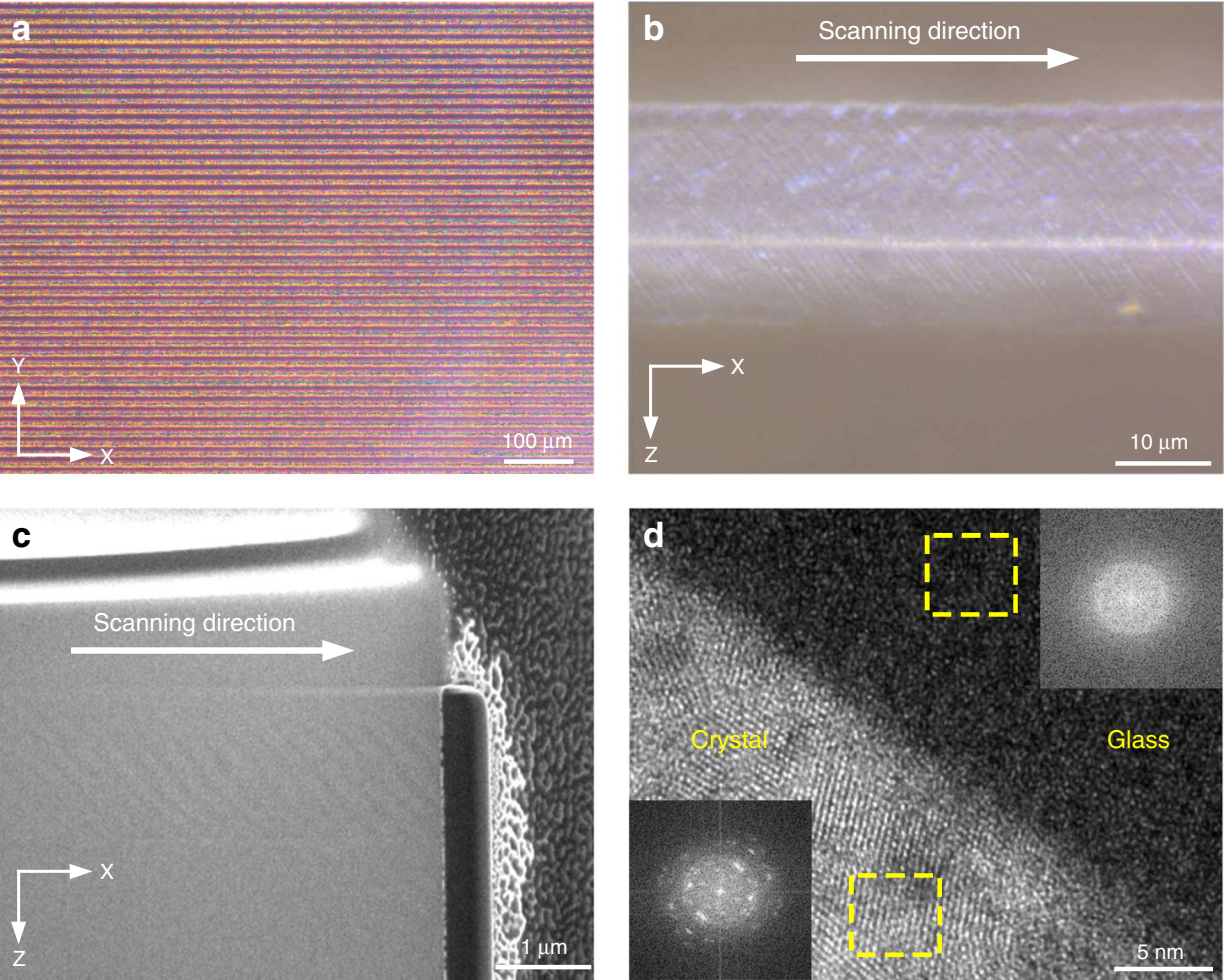
Structural demonstration of the textures. **a** Birefringence image of largely produced photonic textures in the glass. **b** Optical microscope image of the embedded glass-crystal arrays without any mechanical damage. **c** Focused ion beam (FIB) slice of the periodic glass-crystal arrays of the texture. **d** HRTEM image of the glass and crystal region of the texture. Insets are the fast Fourier transform (FFT) images of the dotted areas

We presented the fast creation of large-area photonic textures with fine spatial morphology in the glass and demonstrated their phase-transition-based structural properties. As shown in Fig. 3a, the presented laser-modified area, filled with the textures, reaches millimeter scale and the typical writing speed can be as high as 2 mm s^−1^, which shows that the SOPTL possesses a strikingly higher processing efficiency than conventional techniques. Meanwhile, the uniform and intense artificial birefringence signal in the laser-modified area confirms that the SOPTL has a substantial degree of repeatability and robustness against various external perturbations. Figure 3b displays an optical image of the periodic structures without mechanical damage, and highly regular nanometer-scale textures embedded in the glass matrix can be observed. This ensures a remarkable optical performance of the created structures. Figure 3c shows that the created texture is characterized by the presence of periodically assembled bright- and dark-contrast regions along the beam-writing path. High-resolution transmission electron microscopy (HRTEM) confirms that the periodic textures are essentially alternately arranged crystal-glass phases (Fig. 3d), which is in line with the expected products of the SOPTL. The crystallization structure in the textures is also confirmed by the Raman spectra, as shown in Supplementary Fig. S6. Notably, the feature size of the photonic textures is at around 200 nm (Supplementary Fig. S4a), about 1/4 of the optical diffraction limit (~800 nm) of the beam-writing system (1030-nm laser and 0.8 NA objective), demonstrating the super-resolution machining capability of the SOPTL. By utilizing the differences in optical properties between the glass and crystal phases, these well-organized textures hold potential as photonic elements.

**Fig. 4:**
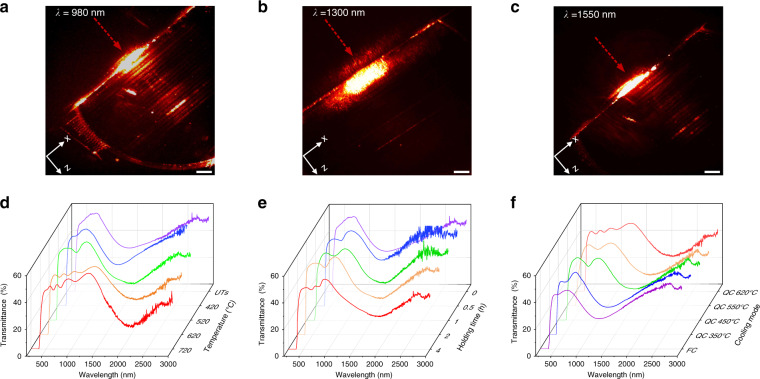
Optical properties of the textures. **a**–**c** Propagation behaviors in multilayer photonic textures of different probe lights with wavelengths of 980 nm (**a**), 1300 nm (**b**), and 1550 nm (**c**), respectively. The red dotted arrows indicate the direction of the incident probe light. Scale bar: 200 µm. **d**–**f** Dependence of transmittance spectra of the photonic textures on different holding temperatures with a holding time of 2 h (**d**), different holding times with a holding temperature of 520 °C (**e**), and different cooling modes (**f**). QC and FC indicate quench cooling and furnace cooling, respectively. For example, QC 450 °C means performing quench cooling at 450 °C

We have characterized the optical properties of the photonic textures. The probe light with various wavelengths of 980 nm (Fig. 4a), 1300 nm (Fig. 4b), and 1550 nm (Fig. 4c) exhibits different transmittance in a sample with multilayer textures along the propagation direction of the writing beam (*Z* axis), and the light at 1300 nm is found forbidden. The transmittance spectrum shows the presence of an obvious broadband transmission reduction in the range of 1000–2000 nm with a center wavelength at around 1300 nm (Supplementary Fig. S5a), indicating the appearance of a broad PBG at near-infrared waveband. The periodic distribution of the refractive index in the crystal-glass textures accompanied with the continuously varied period with the depth is responsible for the spectra modulation and broadness. Besides, a polarization-dependent light attenuation effect in the near-infrared region is also observed (Supplementary Fig. S5b). Considering the spatial morphology of the photonic textures (Supplementary Fig. S5c), the binary optical properties can be understood: for the incident probe light along the *Z* axis, the component propagating perpendicular to the crystalline stripes is modulated by periodic crystal-glass interfaces, and specific wavelengths are selectively reflected (Supplementary Fig. S5d), forming a PBG. Meanwhile, the component that propagates along the crystalline stripes is also affected by the periodic structures. In this case, the periodic crystal-glass arrays are equivalent to the subwavelength gratings (SWGs), which are polarization-sensitive and act as barriers to the incident light with polarization perpendicular to the grating stripes^36,37^. This well explains the coexistence of the PBG and the polarization-dependent attenuation effect.

We demonstrated effective tailoring of the optical properties of the SOPTL-fabricated photonic textures. As an example, introducing a reversible secondary-phase transition of the crystal zones in the textures allows for tuning the refractive index contrast between the amorphous phase and the crystal, providing simple and facile access to manipulate optical performances of the photonic textures. Herein, a heat treatment is adopted to induce phase transition along with a quenching process to retain the high-temperature phase. Figure 4d indicates that the PBG central wavelength of the texture significantly red-shifts from ~1300 to ~2000 nm with the temperature increasing from 420 to 720 °C. Notably, no clear difference is found in the transmittance spectra of the initial untreated sample (denoted as UTs) and that treated at 420 °C, indicating that no structural variation occurs in the crystal regions during heat treatment at 420 °C. Figure 4e demonstrates that an increase in holding time at a fixed temperature of 520 °C can induce a redshift in the PBG, which is in line with the reported secondary-phase-transition temperature^38,39^. This structural variation is further confirmed by Raman spectroscopy (Supplementary Fig. S6). Similar to the PBG, the polarization-dependent attenuation of the photonic textures is broadly manipulated by the heat treatment process. With an increase in the holding temperature, the wavelength with the maximum attenuation ratio red-shifts from ~1100 to ~2000 nm (Supplementary Fig. S7b). Since the secondary-phase transition is reversible^38^, the tuned optical properties of the photonic textures can be easily reset (Fig. 4f). This can be demonstrated by a two-step process that combines furnace cooling and quench cooling, as quench cooling retains the high-temperature phase and furnace cooling gives the room-temperature phase. For example, after 2 h of heat treatment at 620 °C, the temperature slowly decreases to the set value (*T*_f_) with furnace cooling, which is followed by quench cooling to room temperature. For the samples with *T*_f_ of 550 °C and 450 °C, blue-shifts of ~200 nm and 400 nm to a new waveband in the PBG can be identified, respectively, in comparison with the sample quenched directly from 620 °C. A complete furnace cooling can turn the PBG of a heat-treated sample to the initial state. These results demonstrate that phase-transition engineering serves as a simple and effective method to adjust and reset the optical performances of the photonic textures in a considerable range, making the SOPTL-created textures much more flexible and advantageous over conventional photonic structures.

**Fig. 5:**
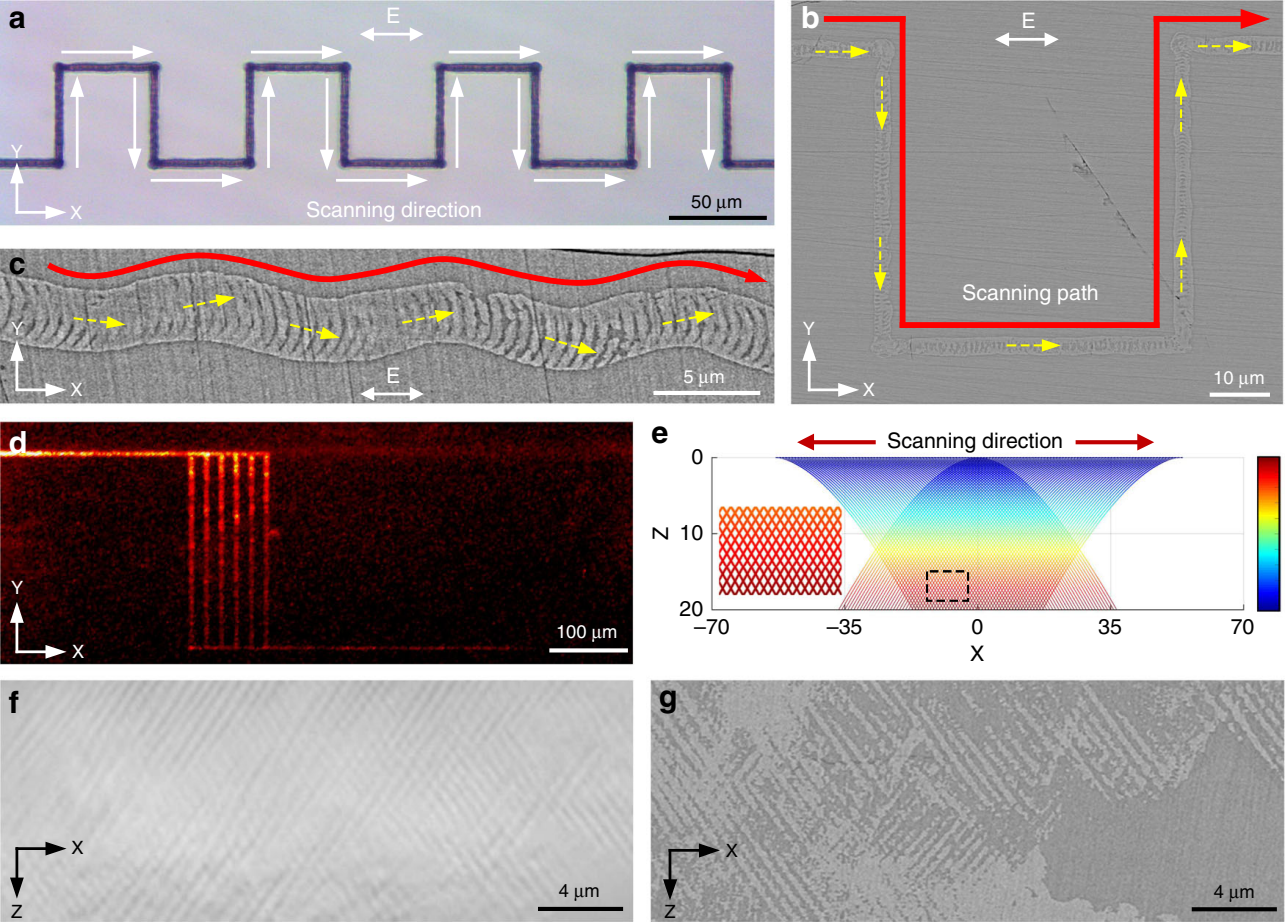
Demonstration of more complex textures. **a** Optical image of the line created by the noncontinuous variable-direction writing process. The direction changes 90° each time. **b** Corresponding SEM image of the textures in the scanning path presented in **a**, and **c** shows an SEM image of the textures induced by the continuous variable-direction writing process. The red line indicates the scanning path and the yellow dotted arrow indicates the orientation of the textures. The laser polarization is denoted by E. **d** Demonstration of waveguides made of the textures using a 980-nm laser. **e** Theoretical morphology of the interlaced texture predicted by the SOPTL principle. Inset: the amplified image of the dotted area. The color bar illustrates the radius change of the EPSs. **f** Optical microscope image of the interlaced textures created by a dual writing process. **g** SEM image of the interlaced photonic textures

The processing mechanism of the SOPTL can be further developed to realize more complicated structural tailoring of the photonic textures. For instance, according to the theoretical model of the SOPTL discussed above, the orientation of the periodic arrays depends on the wave vector of the incident light, which has a component in the scanning direction. This indicates that the texture is scanning-direction-dependent and confers the structure another manipulation degree of freedom. As shown in Fig. 5a, b, in the 90-degree variable-direction writing process, the orientation of the texture only changes with the direction and is independent of the laser polarization (Supplementary Fig. S4b). Further continuous variable-direction writing experiments confirm that such scanning-direction-dependent texture can automatically and continuously adjust its orientation according to the scanning path (*XY* plane, Fig. 5c), which is highly valuable for constructing spatial photonic crystal elements. For instance, it can be applied to form photonic waveguides to allow the light with wavelengths out of the PBG to travel in the designated paths. Figure 5d reveals that the light signal (980 nm) can survive after passing twelve right angles, indicating excellent light-guiding performance. In addition, the textures with different inclinations can theoretically be superposed together by scanning the ultrafast laser beam back and forth (a dual writing process), resulting in interlaced photonic textures in the glass (Fig. 5e). This is well demonstrated by experiments. Figure 5f, g demonstrates the formation of a textured nanostructure that consists of two sets of interlaced periodic structures tilted in different directions and this is nearly impossible to achieve by any other approaches. The periodic crystal-glass textures in both *X* and *Z* axes can serve as 2D PCs working in the near-infrared region. In principle, embedded 3D PCs can be further realized by a multilayer SOPTL process.

## Discussion

Controlling the interference-induced periodic crystallization behavior is the intrinsic mechanism of the SOPTL. Therefore, manipulating the laser parameters and the chemical composition of the glass matrix holds the potential to tune the structural and optical properties of the photonic textures. For example, our experiments indicate that the inclination degree of the crystal-glass arrays can be manipulated by adjusting the numerical aperture of the objective (Supplementary Fig. S8a, b). Pulse energy also has an effect on the spacing of the ripples. This effect is reflected in the degree of crystallization of the glass matrix. As higher laser fluence will lead to a stronger thermal effect in the interference field, the spacing of the ripples may shrink or even disappear, which is firmly confirmed by our experiments (Supplementary Fig. S8d). Besides, by gradually increasing Ta_2_O_5_ content in the LTN glass, the PBG can be considerably enlarged and becomes much more visible (Supplementary Fig. S7a). Therefore, properly designing laser parameters and glass composition used for the SOPTL offers a direct and efficient way to refine the optical characteristics of the texture.

More importantly, we must emphasize that the proposed physical model can be applied to other glass/crystal materials and similar periodic structures have been successfully created in several commonly used transparent dielectrics, such as fused silica, quartz, and sapphire, which has been confirmed by our experiments (Supplementary Fig. S9). This indicates that the proposed physical model possesses a considerable degree of universality and can serve as a general principle for fabricating all-inorganic photonic structures in different transparent media. In addition, the produced textures are well protected by the glass/crystal matrix around, which makes them possess a remarkably long service life and are highly immune to external environmental disturbances such as heat, pressure, moisture, and pollution^40,41^.

In conclusion, the reported 3D interference actively induced by ultrafast laser–matter interaction in the focal area presents a novel optical field modulation and a universal super-resolution tool to produce complicated photonic textures in transparent dielectrics using a single step, breaking through the technical hurdles in achieving single-beam interference lithography. The proposed scattering-interference model provides convincing descriptions to all experimental phenomena and further predicts important properties of the SOPTL process, which allows for the construction of more complicated 3D photonic structures and control over their structural features. The generated photonic textures possess a broad PBG in the near-infrared region, which can be tuned in a wide range by engineering a secondary-phase transition. More importantly, only a few seconds are required to fabricate large-area photonic textures with a nanometer-level resolution, indicating the promise of the SOPTL in overcoming the bottleneck in the cost and efficiency faced by current nanofabrication techniques. It would be exciting to combine the current laser processing techniques with SOPTL to develop a general technique to achieve in-chip photonic devices at the on-demand position in transparent dielectrics, which will open up brand-new routes for a variety of frontier photonic technologies, including asymmetric gratings, environmental sensors, photonic crystals, dispersion elements, and nonlinear optics.

## Materials and methods

### Materials

In this study, the textures were created in a multicomponent LTN glass, which is made from a mixture of lanthanum oxide, niobium oxide, and tantalum oxide raw powders at a ratio of 35La_2_O_3_–*x*Ta_2_O_5_–(65-*x*)Nb_2_O_5_ (where *x* = 0, 15, 25, and 35 mol%). The glass for the SOPTL was prepared by a containerless process where two CO_2_ lasers were used to melt the mixture of raw materials levitated by the O_2_ airflow. Before the SOPTL, the blank glass samples were milled into sheets and polished.

### Ultrafast laser processing

In the ultrafast beam-writing process, a mode-locked regeneratively amplified Yb:KGW-based ultrafast laser system (PHAROS, Light Conversion Ltd.) operating at a wavelength of 1030 nm with a pulse energy of 1 µJ, a pulse duration of 1 ps, and a repetition rate of 150 kHz was employed as the light source. Generally, the laser beam was tightly focused ~60 µm below the surface of the sample via a ×50 objective lens (NA = 0.8). In multilayer SOPTL, the processing depth can be up to ~500 µm. The beam writing was performed by moving the samples at a speed of 2 mm s^−1^, and the highest scanning speed can be up to 6 mm s^−1^. The process window for the SOPTL process is shown in Supplementary Fig. S1b. The movement of the samples in *XYZ* axes was realized by a computer-controlled micropositioning platform (Supplementary Fig. S1a).

### Testing methodology

Optical microscopy was performed by a polarizing microscope. The structural properties of the samples were examined by field-emission scanning electron microscopy (FE-SEM), using backscattering mode. For the FE-SEM observation, the textures were polished so as to expose the laser-irradiated region to air and the polished surface was then etched by hydrofluoric acid to improve the contrast of FE-SEM images. Further structural characterization of the created texture was performed by transmission electron microscopy (TEM). The transmittance spectra of samples were measured using a UV–visible NIR spectrophotometer and the secondary-phase transition in the textures was demonstrated by a laser confocal micro-Raman (LCM-Raman) spectrometer excited by a 532-nm diode-pumped solid-state (DPSS) laser. The heat treatment process was performed in a tube furnace.

Experimental characterization of the light attenuation effect of the textures was performed by illuminating the sample along the *Z* axis with probe lights that have different polarizations and wavelengths and measuring the variation-transmitted light power (Supplementary Fig. S1c). The glass sample was filled with the lines that consist of the textures. The interval of the beam-writing paths is ~15 µm. The near-infrared light propagation behavior in the textures was imaged using a thermoelectrically cooled InGaAs camera sensitive in the wavelength range 0.9–1.7 µm.

## Supplementary information

Supplementary information

A animation describing the dynamic process of the photonic texture formation
