# Supplementary material for: Self-organized phase-transition lithography for all-inorganic photonic textures
Source: Light Sci Appl. 2021 Apr 29;10:93. doi: 10.1038/s41377-021-00534-5 (PMC8085003; doi:10.1038/s41377-021-00534-5)
Supplement: Supplementary file 1 — Supplementary information [file 41377_2021_534_MOESM1_ESM.docx]

Supplementary Information for

**Self-organized phase transition lithography for all-inorganic photonic textures**

Bo Zhang^1†^, Dezhi Tan^1*†^, Zhuo Wang^1^, Xiaofeng Liu^2^, Beibei Xu^1^, Min Gu^3^, Limin Tong^1^, Jianrong Qiu^1,4*^

^1^State Key Laboratory of Modern Optical Instrumentation, College of Optical Science and Engineering, Zhejiang University, Hangzhou 310027, China

^2^School of Materials Science and Engineering, Zhejiang University, Hangzhou 310027, China

^3^Centre for Artificial-Intelligence Nanophotonics, School of Optical Science and Engineering, Shanghai University of Science and Technology, Shanghai 200093, China

^4^CAS Center for Excellence in Ultra-intense Laser Science, Chinese Academy of Sciences, Shanghai, 201800, China

*Correspondence to: wctdz@zju.edu.cn & qjr@zju.edu.cn

†These authors contributed equally to this work.

**Contents**

S1 Experimental setups

S2 Derivation of the single scattering center model

S3 Structural characterization of the photonic textures

S4 Optical characterization of the photonic textures

S5 Raman characterization of the secondary phase transition

S6 Optical and structural manipulation of the photonic textures

S7 Demonstration of the universality of the self-organized phase transition lithography (SOPTL) principle

### S1 Experimental setups

As shown in Fig. S1a, a laser direct writing system is used to activate the SOPTL process and fill a bulk La_2_O_3_-Ta_2_O_5_-Nb_2_O_5_ glass (LTN glass) sample with lines consist of the textures. Fig. S1b shows the process windows for the texture fabrication. The experimental configuration for testing the polarization-dependent light attenuation effect of the textures is shown in Fig. S1c.


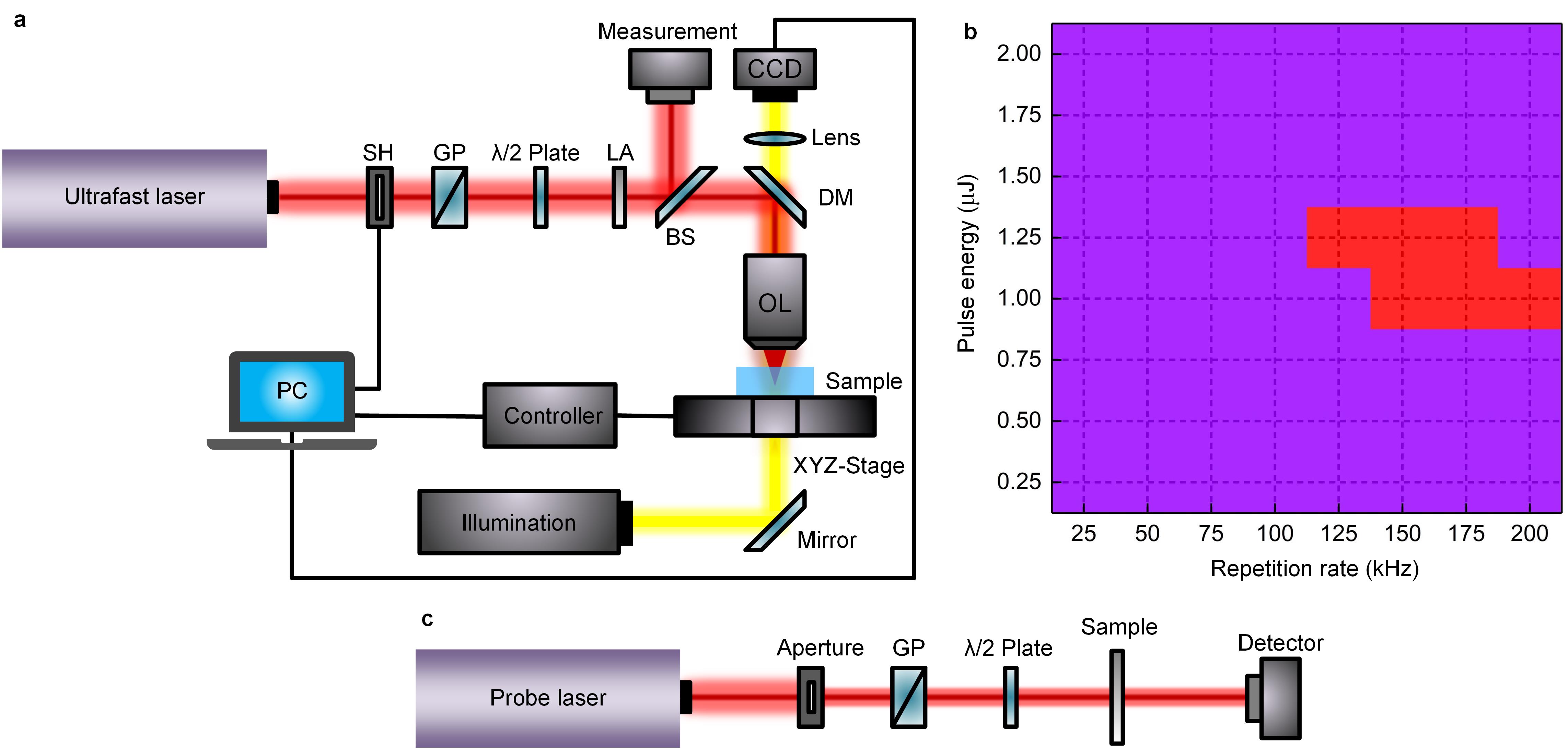


**Fig. S1 Experimental setups and parameters. a** The ultrafast laser direct writing system for inducing the textures. SH: shutter. GP: Glan prism. LA: light attenuator. BS: beam splitter. DM: dichroic mirror. **b** Process window with respect to repetition rate and pulse energy for the texture fabrication. Red region shows the conditions at which the texture can be formed (pulse duration: 1 ps). **c** The schematic diagram of the experimental configuration for testing the light attenuation effect of the textures.

### S2 Derivation of the single scattering center model

As a brand new phenomenon of ultrafast light-matter interaction, no theory, so far, has been proposed to interpret the formation of such periodic glass-crystal textures. In our case, three experimental details provide important clues for understanding the texture formation mechanism. First, static irradiation and local crystallization are necessary before starting the SOPTL processing. Second, the inclination degree of the textures is gradually varied rather than a constant, resulting in clear arcuate stripes in the XZ plane, and seems to follow specific mathematical functions. Third, the textures exhibit an obvious directionality that depends on the beam scanning direction. In summary, two basic requirements must be fulfilled before the SOPTL can be realized: a well-defined interference field and a proper crystallization condition.

First of all, to obtain a well-defined interference field, it is important to have clear and definite light sources that participate in the interference. It is well known that the ultrafast light-matter interaction will modify the material through multi-photon ionization. Meanwhile, the transparent medium used for the SOPTL is La_2_O_3_-Ta_2_O_5_-Nb_2_O_5_ glass (LTN glass) which possesses a stronger tendency to crystallize compared to conventional glasses. The multi-photon ionization at the focus will create plasmas that can generate a high temperature to locally crystallize the glass matrix at the focus, creating a scattering center. This process needs a certain amount of incident pulses, which well explains why static irradiation is needed before the SOPTL.

Enlighten by the well-defined curved stripes in the XZ plane, we further abstract the mechanism for the SOPTL to a single scattering center interference model to describe the texture formation: accompanied with the laser irradiation, a local crystallization zone is gradually formed at top of the focal area and becomes a scattering center for the incident light. The oblique incident light (plane wave) generated by the focusing effect of the objective lens will interfere with scattered light (spherical wave) from the scattering center.


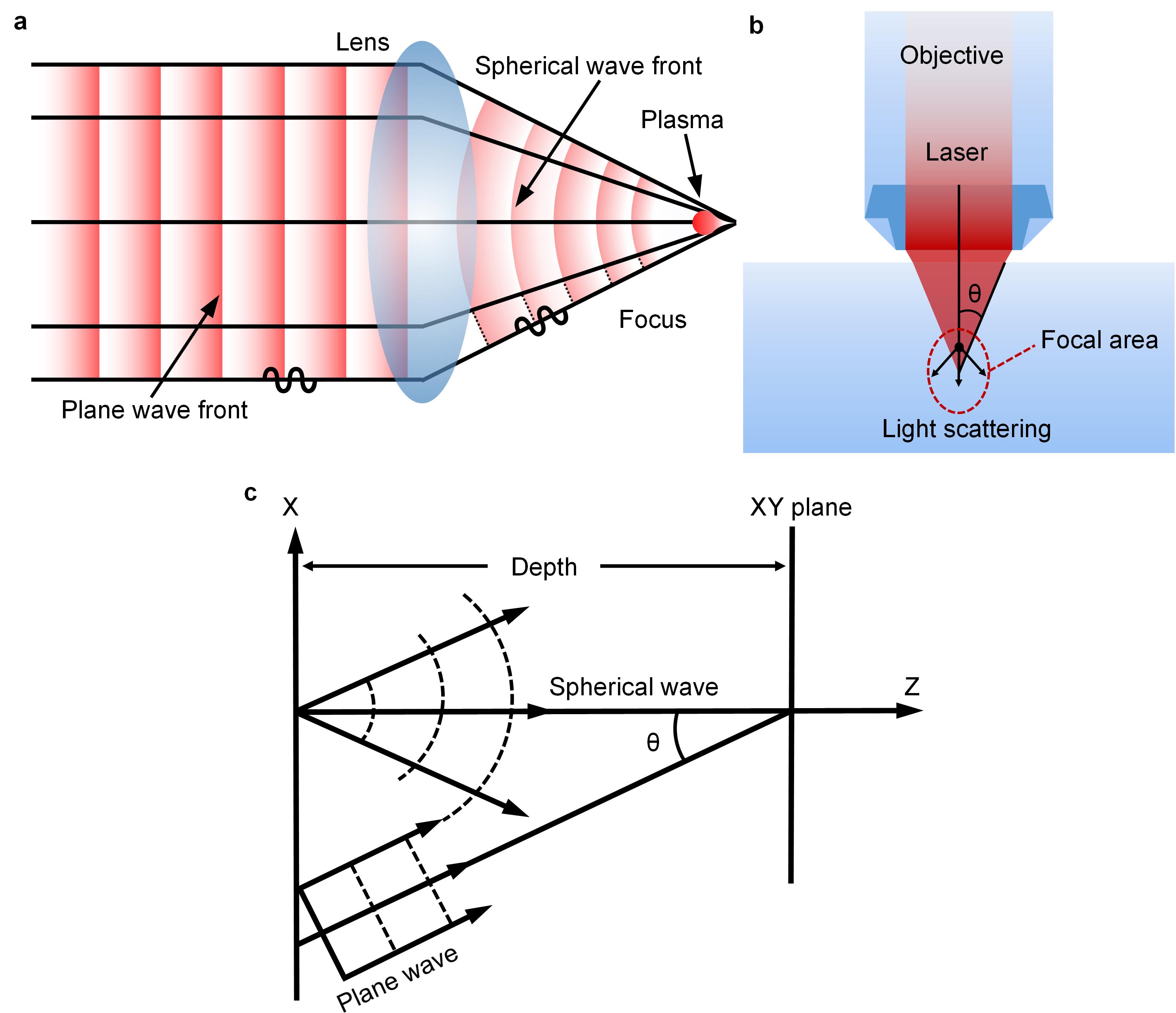


**Fig. S2 Demonstration of the light interference field in the focal area abstracted from the single scattering center model.** **a** Schematic diagram of the plane wave approximation. **b** The schematic diagram of the light propagation model in the glass sample after focused by an objective lens. **c** The interference field of the single scattering center model in the established Cartesian coordinate system.

In general, after focusing, the laser beam will become a convergent spherical wave with the focal point as the center of the sphere. However, due to the nonlinear ionization effect of ultrafast laser-matter interaction[^1^](#_ENREF_1), most part of the focused ultrafast light will be absorbed by the medium and generate a large number of free electrons, forming plasma at the focal area[^2^](#_ENREF_2), which is the origin of the scattering center. Only a small part of the light distributed at the periphery of the focused beam whose intensity is lower than the material's nonlinear absorption threshold can continue to propagate (its wave front is indicated by the dotted line in Fig. S2a). In this condition, the local curvature of the wave front is small enough to be regarded as a plane wave. Namely, the spherical wave is the overall characteristics of focused light, and the plane wave is the local characteristics of the light that participated in the texture formation. In fact, we use the outermost light to simulate the interference field. The incident angle of the plane wave is set as the maximum convergence angle of the objective lens (Fig. S2b), which is the rationale of the structural tilt of the textures.

In order to calculate the interference field distribution, the Cartesian coordinate system is established as shown in Fig. S2c. The scattering center is set as the origin with the depth of zero in the Z-axis and the focused incident light angles *θ* to Z-axis. Here, *θ* is dependent on the numerical aperture (NA) of the objective lens in the processing system and the refractive index of the glass (Fig. S2b). In the established coordinate system, the depth of the interference field is represented by the Z-axis location, and the top of the interference field is prescribed as the origin. The laser propagation direction is prescribed as the positive direction of the Z-axis. In this way, the complex amplitude of the oblique incident light and the scattered light can be expressed as:

$\left\{ \begin{aligned} \tilde{E}_{i}\left( x,y \right)={E_{i}e}^{-ikxsin\theta} \\ \tilde{E}_{s}\left( x,y \right)={E_{s}e}^{ik\frac{x^{2}+y^{2}}{2z}} \end{aligned} \right.$ (S1)

Where *k* is the wave vector, $\theta$ is the angle between the oblique incident wave vector and *Z*-axis depending on the glass refractive index (RI) and the NA of objective, *i* is the imaginary number, and $E_{i}$ and $E_{s}$ are the light vectors of incident light and scattered light representatively. Considering the incident light closed to the optic axis will be then scattered by the scattering center in the focal area, we believe that only the outermost oblique incident light participates in the interference with the scattered light. The inclination angle $\theta$ satisfies the relationship: $nsin\theta=NA$, where *n* is the RI of the LTN glass. According to the spatial light interference principle, the light intensity $I\left( x,y \right)$ in the interference field can be derived as:

$I\left( x,y \right)={E_{i}^{2}+E_{s}^{2}+2E_{i}E}_{s}cos\left[ k\left( \frac{x^{2}+y^{2}}{2z}-xsin\theta\right) \right]$ (S2)

For a certain depth below the scattering center (denoted as *z*), the fringes of interference enhancement can be expressed as follows:

$\left\{ \begin{aligned} R^{2}=y^{2}+{(x-zsin\theta)}^{2} \\ R=\sqrt{z^{2}\sin^{2} \theta+2\lambda mz} \end{aligned} \right.$ (S3)

Where *R* is the radius of the interference fringes, $\lambda$ is the light wavelength (1030 nm) and *m* is the inference order (*m* = 0, 1, 2, 3, 4….). Therefore, we can find that the spatial distribution of the interference field is actually a series of equal-phase surfaces (EPSs) of constructive interference, and the seen periodic structure is corresponding to the intersecting patterns between the EPSs and observation planes.

According to the calculated interference fields, the variation of the period of the textures as a function of the depth and the interference order (*m*) can be deduced by the radius difference $\Delta R$ between two neighboring EPSs:

$\Delta R=\sqrt{z^{2}\sin^{2} \theta+2\lambda(m+1)z}-\sqrt{z^{2}\sin^{2} \theta+2\lambda mz}$ (S4)

The curve extracted from the edge of the equal-phase face outline in the XZ plane can be expressed as:

$R=\sqrt{z^{2}\sin^{2} \theta+2\lambda mz}+z\sin\theta$ (S5)

Where $\lambda$ is the laser wavelength and $z$ is the Z-axis location (depth) of the investigated interference field. Now, we understand that all the experimental observations, including the varied bending degree with the depth, arc-shaped stripes in the XY plane, and tilted periodic structures, mathematically follow the calculated EPSs. By this point, all the important phenomena of the textures are persuasively explained and quantitatively described, proving the validity of the single scattering center theory.


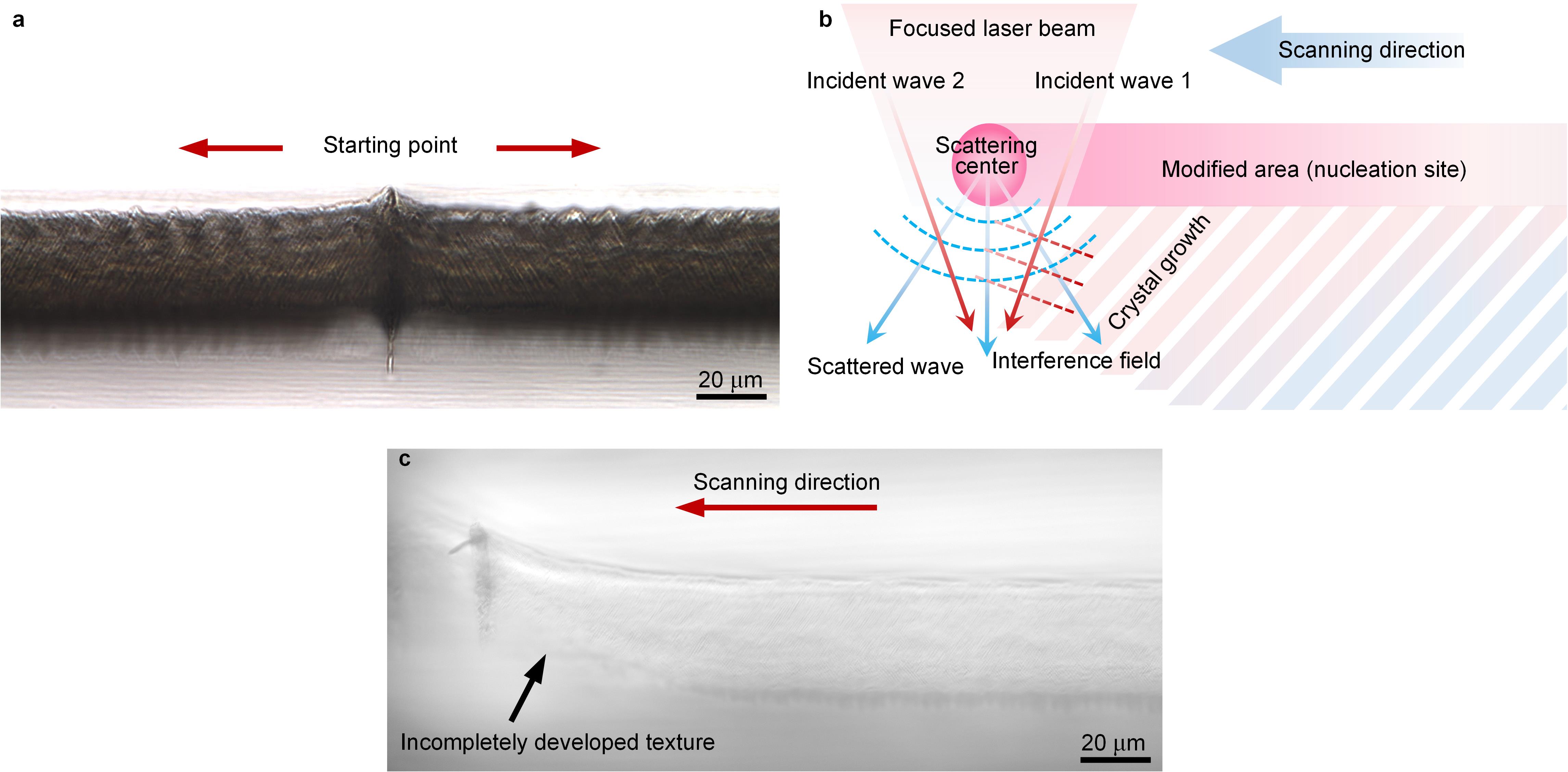


**Fig. S3 The directionality of the texture and crystal growth in the SOPTL. a** Optical microscope image of the side view of two lines independently written with opposite scanning directions (indicated by the red arrows) from the same starting point. **b** Schematic diagram of the crystal growth in the SOPTL process based on the single scattering center model. **c** Incomplete crystal growth in the SOPTL process when there is a scanning shutdown.

Based on the third clue, the texture formation shows an obvious directionality, precisely depending on the beam scanning direction, we can preliminarily reveal the formation mechanism of the textures. Experimental results indicate that only the EPSs that incline toward the starting point can participate in the SOPTL process (Fig. S3a). In other words, the interference field, created by the incident light that propagates through the modified area (incident wave 1 in Fig. S3b), actually comes into play in the texture formation. This demonstrates that only creating the well-defined interference field is not enough for achieving the SOPTL and a proper crystallization condition is also needed. We can infer that the laser modified region, at the top of the interference field and behind the focus, provides a suitable nucleation site and a temperature gradient for crystal growth. Then, the crystal part of the texture emerges from the top nucleation area and grows along the specific EPSs, resulting in the scanning-direction-dependent periodic structures. Limited by crystallization conditions, the interference fields of the incident lights that do not propagate through the modified area (incident wave 2 in Fig. S3b) cannot induce the crystallization process, which well explains the directionality of the texture formation as well as its inclination towards the starting point.

In view of these results, we can briefly outline the texture formation process. The static irradiation at first is to generate a local micro-crystallization area that serves as a scattering center and nucleation sites in the early stage of the SOPTL processing. Then the interference of the oblique incident plane wave and the scattered spherical wave will create an interference field, leading to periodic temperature gradient distribution. Finally, the crystal stripes emerge from the nucleation zone and grow along the EPSs of constructive interference in the interference field, resulting in the textures (movie S1). This process is a little lagging behind the laser scanning movement and can be verified by abruptly interrupting the SOPTL process and checking the side view of the textures. As shown in Fig. S3c, we can clearly see the incompletely developed texture at the position where the scan line is interrupted, confirming the accuracy of the proposed theoretical model.

Furthermore, in principle, the interference between the scattered laser light and the main laser beam may be present in a broad space with continuous propagation. In our case, the scattering light (spherical wave) comes from the laser-induced local modification, whose intensity decreases rapidly with the increase of the propagation distance, so that the intensity of the interference field far away from the focus area is lower than the threshold for triggering the glass-crystal phase transition. Therefore, the texture formation only occurs in a very confined space in the focal area. The limited volume of periodic structures (Fig. 2e, h) also confirms that the effective interference field locates in a confined space. In addition, the three-dimensional shape of this structure presents a nearly standard hyperboloid form that is a very typical spatial pattern of the interference field of obliquely incident plane wave and spherical wave (shown in Fig. 2c-h). This supports the validity of the scattering-interference model.

### S3 Structural characterization of the photonic textures


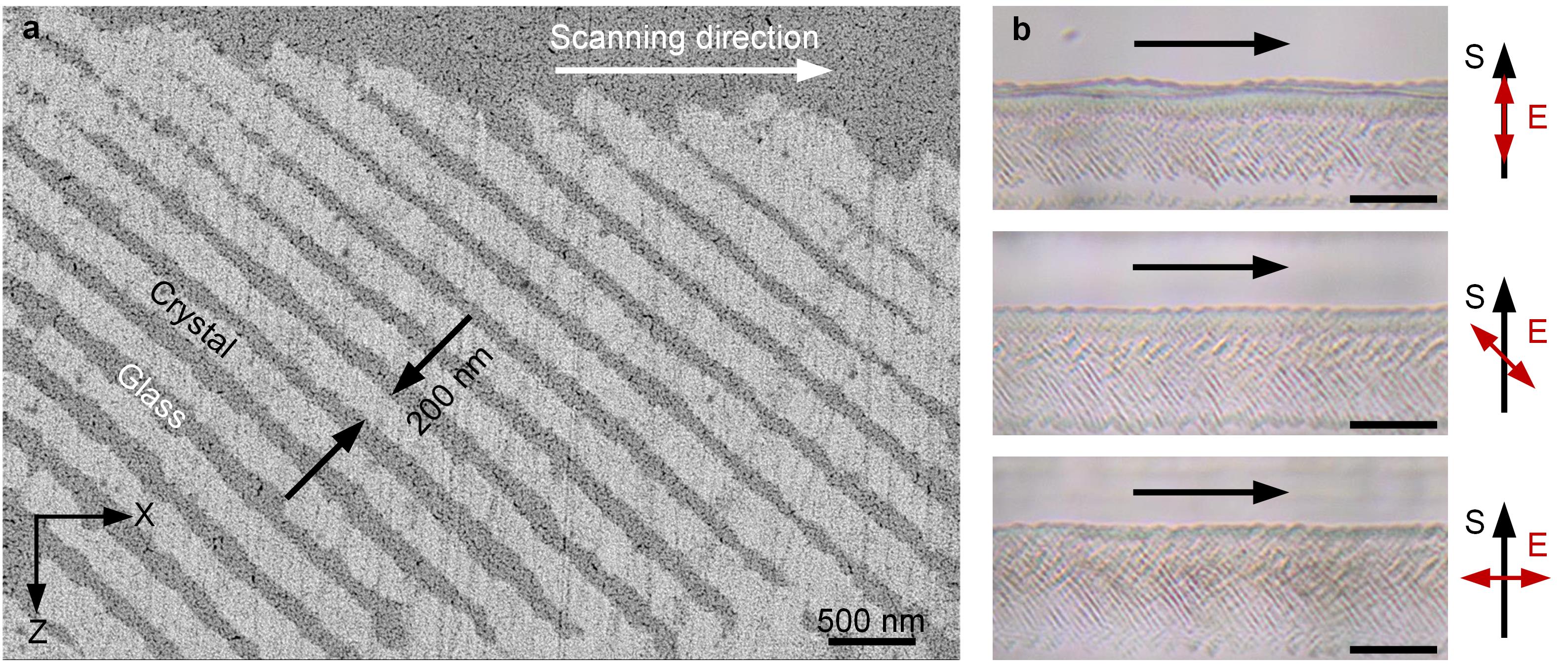


**Fig. S4 Demonstration of the structural properties of the texture. a** Magnified SEM image of the side view of the created textures in the XZ plane. **b** Side view of the textures written by an ultrafast laser with various polarizations. Black arrows indicate the scanning direction (denoted by S) and red arrows indicate the polarization direction (denoted by E). Scale bar: 20 µm.

As the ultrafast light-matter interaction is actually based on the multi-photon nonlinear process, the SOPTL is a super-resolution nanostructuring approach. As shown in Fig. S4a, we demonstrate the super-resolution micromachining capability of the SOPTL technique with ~200 nm feature size, which is about 1/4 of the optical diffraction limit of the laser direct writing system (1030 nm laser and 0.8 NA objective). The created periodic structure in our work is scanning-direction-dependent rather than polarization-dependent, which is very different from conventional periodic structures reported inside transparent media. This is determined by the theoretical model. As shown in Equation S2, there is no light polarization parameter in the derived analytical solution of the interference field. Therefore, the ripple created by this mechanism should be polarization-independent, which is firmly verified by polarization conversion experiments (as shown in Fig. S4b). It can be seen that the side morphologies of the periodic structure written by different polarizations are the same, which indicates that the spatial orientation of the fringes has nothing to do with the polarization of the incident light.

### S4 Optical characterization of the photonic textures


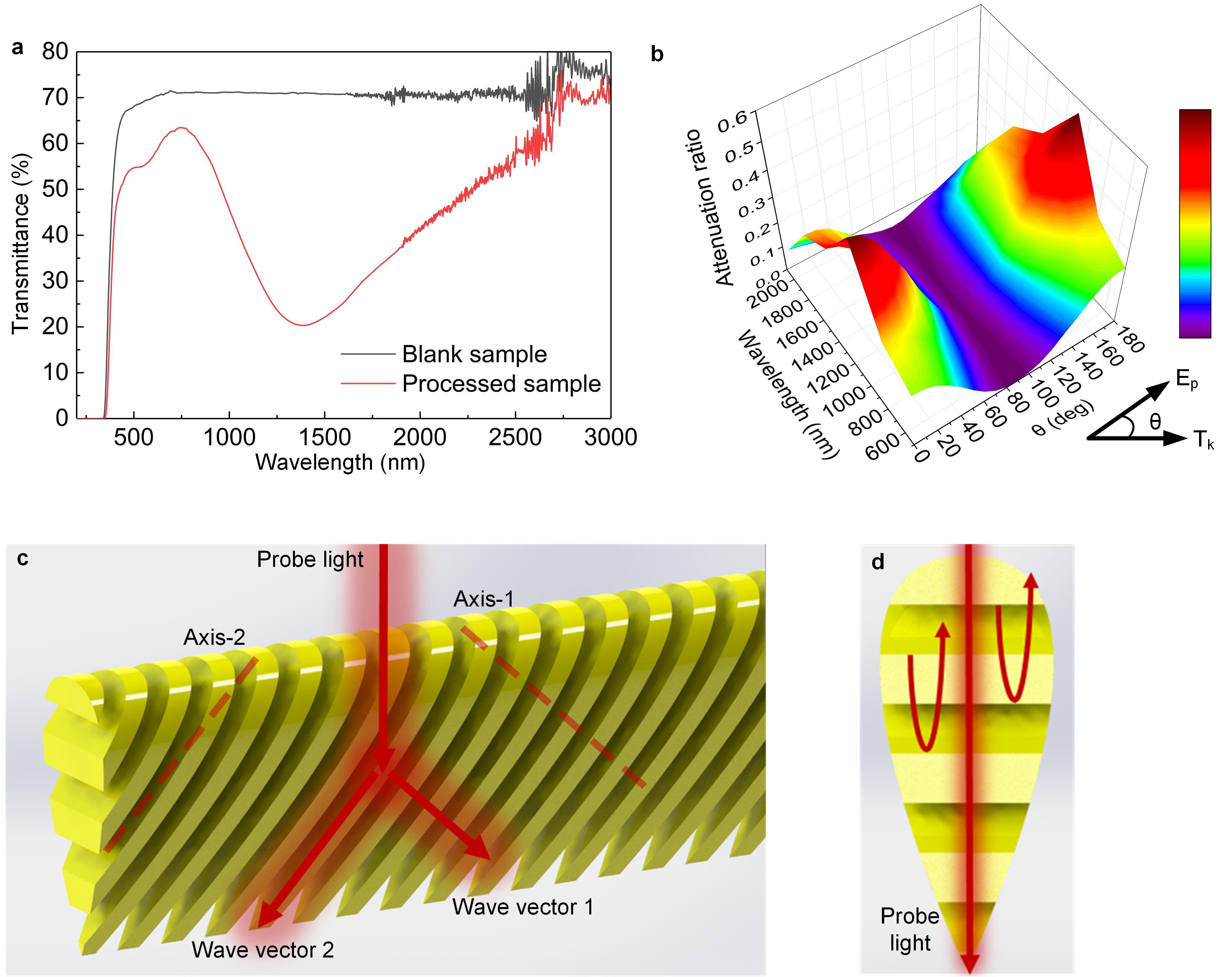


**Fig. S5 The binary optical properties of the created textures. a** Transmittance spectra of blank glass sample and sample filled with the textures written by ultrafast laser. **b** Polarization-dependent light attenuation as a function of the relative angle (indicated by θ) and wavelength. The polarization of the probe lasers is indicated by E_p_ and the orientation of the textures (perpendicular to the slow axis) indicated by T_k_. Color bar shows the attenuation rate. **c** Schematic diagram of light propagation in the texture. **d** Schematic diagram of light wave propagates along the axis-1 of the texture.

The transmission spectra of the laser processed sample and a blank one further indicate that the processed sample exhibits an obvious photonic bandgap (Fig. S5a). And the polarization-dependent light attenuation effect in near-infrared waveband is confirmed by the light attenuation tests (Fig. S5b). Combining with the 3D model of the textures (Fig. S5c), the optical properties of the texture can be understood. The probe light wave propagates along the axis-1 (Wave vector 1 in Fig. S5c) is modulated by periodic glass-crystal interfaces and a certain waveband is selectively reflected (Fig. S5d), forming a photonic bandgap. For the light wave propagates along the axis-2 (Wave vector 2 in Fig. S5c), the periodic glass-crystal arrays are equivalent to subwavelength gratings and the polarization component perpendicular to the grating stripes is selectively prohibited.

### S5 Raman characterization of the secondary phase transition


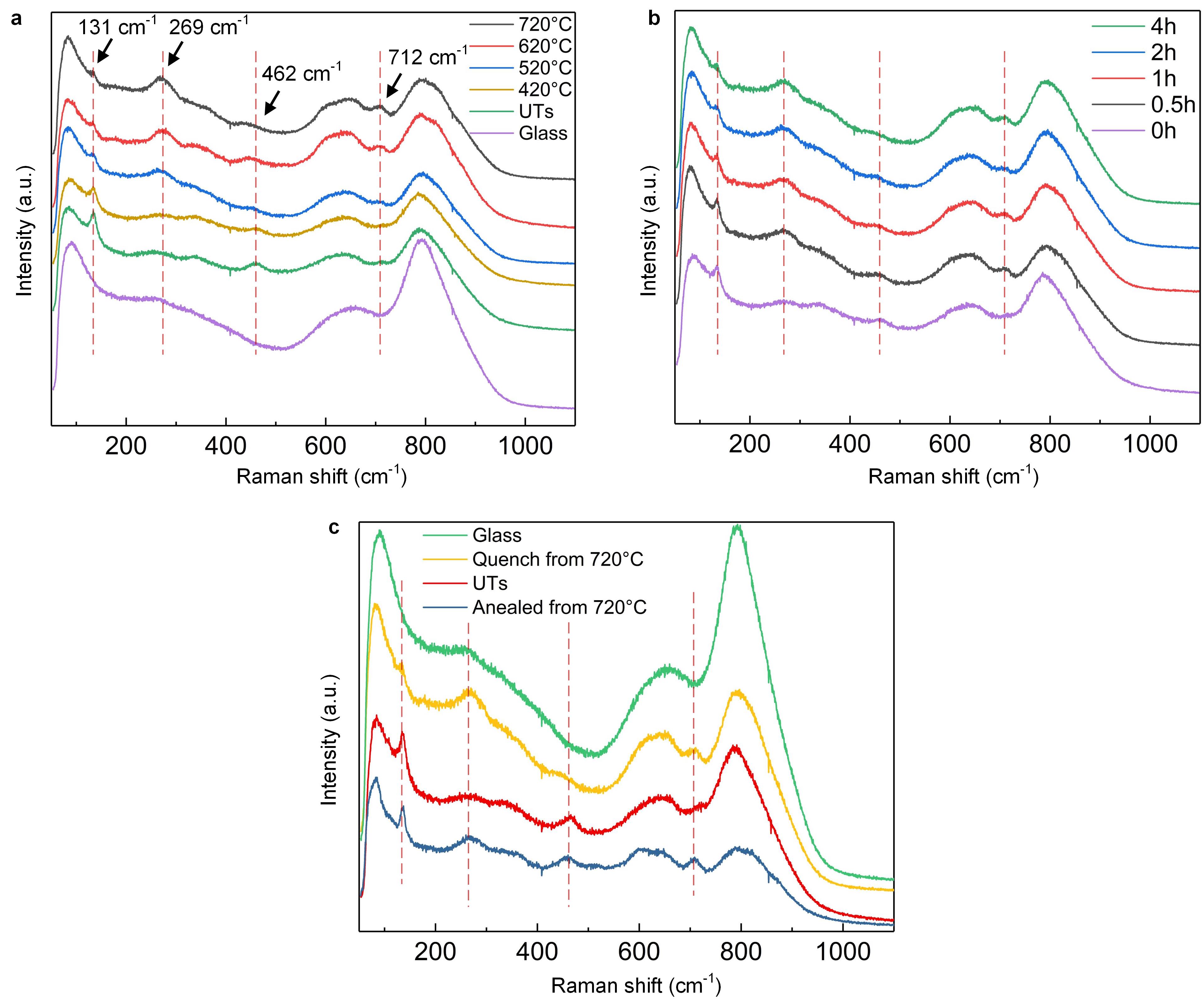


**Fig. S6 Raman characterization of the secondary phase transition.** **a** Dependence of Raman spectra of the processed area on different holding temperatures, compared with the untreated sample (denoted as UTs) and LTN glass. **b** Dependence of Raman spectra of the processed area on different holding times with a holding temperature of 520 °C. **c** Dependence of Raman spectra of the processed area on different cooling modes, compared with untreated sample and LTN glass.

Fig. S6a shows the Raman spectra of processed samples quenched from different holding temperatures, where we can clearly find that the Raman spectrum of a sample quenched from 420 °C (denoted as 420 °C spectrum) is the same as an untreated one (denoted as UTs). This indicates that the phase transition did not happen during the heat treatment process. However, the spectra for the samples quenched from 520 °C, 620 °C, and 720 °C are obviously different from that of the sample quenched from 420 °C and UTs, indicating that the phase transition occurs at a temperature of 520 °C and above, which is in line with the previous studies[^3^](#_ENREF_3)^,^[^4^](#_ENREF_4). More nuanced phase transition progress is given by Raman spectra of the samples with different holding times at 520 °C. As shown in Fig. S6b, with the increase of holding time, Raman peaks at ~131 cm^-1^ and ~462 cm^-1^ gradually weaken, while Raman peaks at ~269 cm^-1^ and ~712 cm^-1^ enhance, which is in agreement with the result that the phase transition occurs at 520 °C. Under this condition, the phase transition can be finished in only 2 hours. The peaks at 131 cm^-1^ and 462 cm^-1^ can be assigned to feature modes of the LaTa_x_Nb_1-x_O_4_ crystal phase which possesses a monoclinic-type fergusonite structure at room temperature[^5^](#_ENREF_5)^,^[^6^](#_ENREF_6). The enhanced Raman peaks at around 269 cm^-1^ and 712 cm^-1^ are reported due to the molecular vibrations of Ta_2_O_5_ crystal[^7^](#_ENREF_7)^,^[^8^](#_ENREF_8), indicating the precipitation of Ta_2_O_5_ crystallites in the processed area. The reversible phase transition process can be observed in the Raman spectra of samples treated with different cooling modes. As expected, it is obvious that the peaks at 131 cm^-1^ and 462 cm^-1^ disappear after quench cooling but can be recovered by furnace cooling after reheating to 720 °C (Fig. S6c). As quench cooling can retain the high-temperature phase while furnace cooling will give a room-temperature phase, this phenomenon strongly supports the point that the crystal part of the texture undergoes a reversible phase transition.

### S6 Optical and structural manipulation of the photonic textures


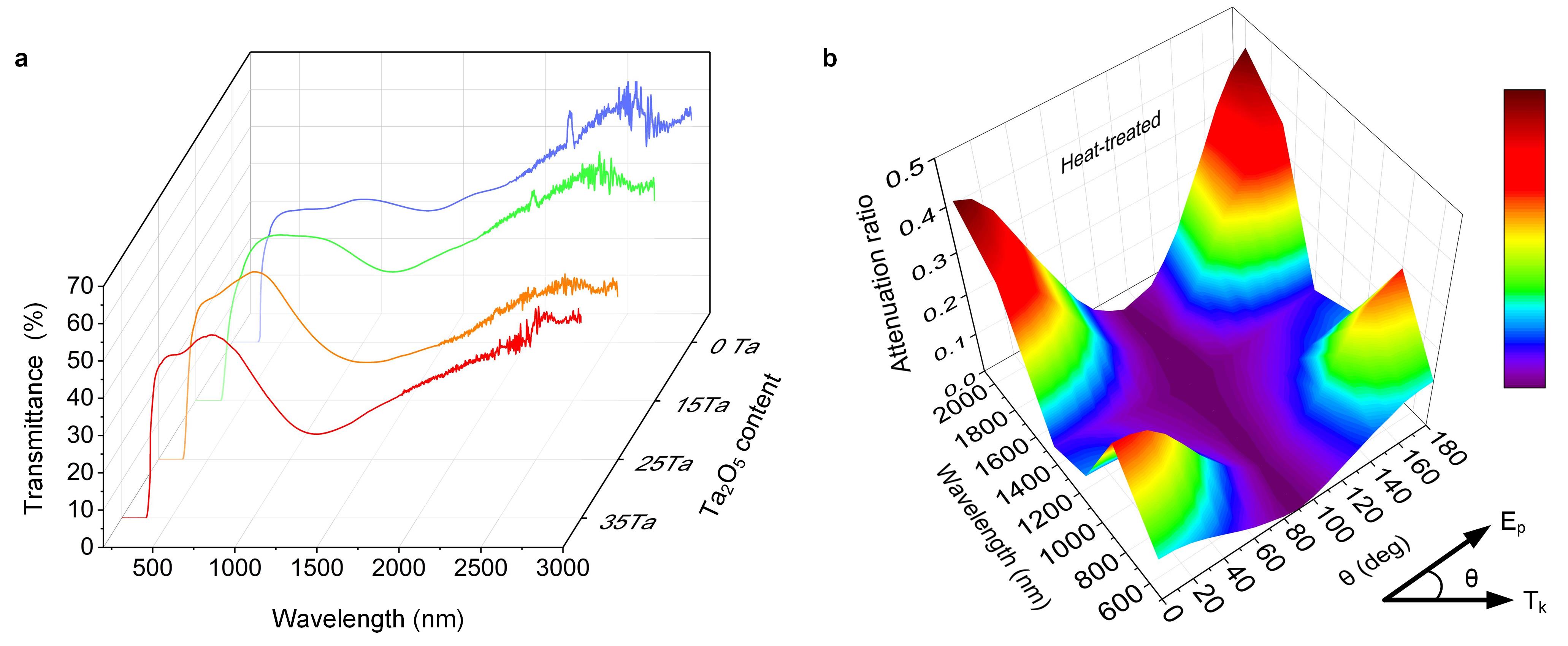


**Fig. S7 Demonstration of optical manipulation of the created textures.** **a** Dependence of transmittance spectra of processed samples on different Ta_2_O_5_ contents from 35 mol% (denoted as 35Ta) to zero (denoted as 0Ta). **b** Polarization-dependent light attenuation effect of sample quenched from 720 °C with a holding time of 4 hours. The polarization of the probe lasers is indicated by E_p_ and the orientation of the textures (perpendicular to the slow axis) indicated by T_k_. Color bar shows the attenuation rate.

Another way to modify the optical properties of the textures is by adjusting Ta_2_O_5_ content. As we have known that Ta_2_O_5_ crystal precipitation in the texture occurs during the SOPTL process, it is reasonable that Ta_2_O_5_ content has an effect on the photonic gap of the textures. This is verified by the transmitted spectra of samples with different Ta_2_O_5_ contents (Fig. S7a). With gradually decreasing Ta_2_O_5_ content to zero, the photonic gap decreases and tends to vanish. The bandgap of the sample with 15 mol% Ta_2_O_5_ content (denoted as 15Ta) is much smaller than the one with 35 mol% Ta_2_O_5_ content. These results are attributed to the important role that Ta_2_O_5_ plays in the texture formation: first of all, Ta_2_O_5_ acts as a crystallization promoter in LTN glasses[^9^](#_ENREF_9), which will motivate the formation of crystal in the texture. Then, the Ta precipitation favors increasing RI contrast between glass phase and crystal phase[^10^](#_ENREF_10), enabling a more significant photonic bandgap. In other words, the addition of a crystallization promoter contributes to the texture formation, which is of guiding significance in designing proper glass systems for the SOPTL.

The polarization-dependent attenuation follows a similar principle as the photonic bandgap: with an increment of holding temperature, the central wavelength of the maximum attenuation ratio significantly red-shifts from ~1100 nm to ~2000 nm (Fig. S7b). These results firmly prove that by introducing thermotropic crystal-to-crystal phase transition, the optical properties of the textures can be effectively regulated in a wide wavelength range, which means that all-inorganic photonic textures can also possess highly flexible working performances.


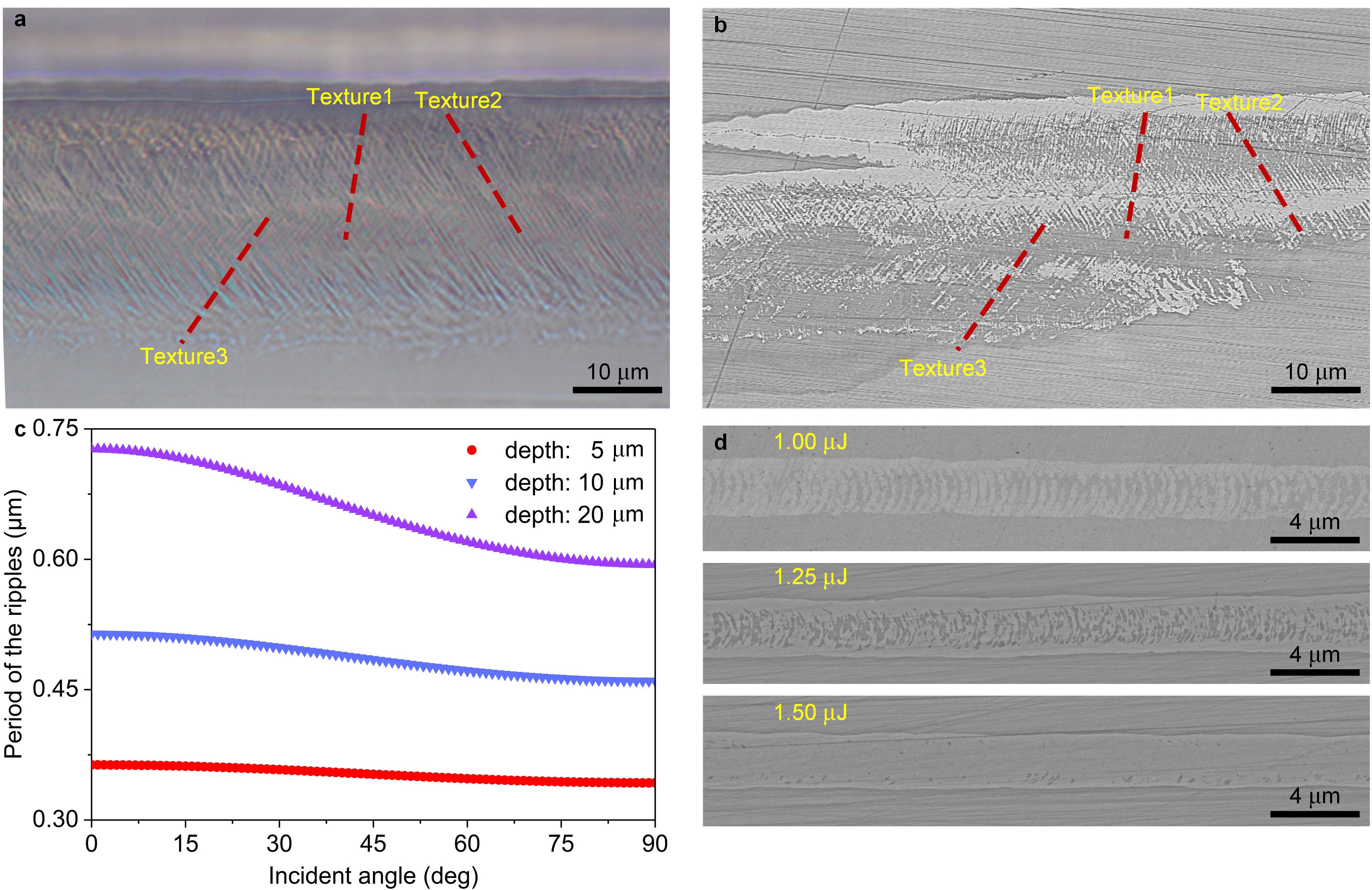


**Fig. S8 Demonstration of the structural manipulation of the textures. a** Optical microscope image of the superposition of three different textures in the glass. Texture 1, Texture 2, and Texture 3 are induced by using 100×1.3, 50×0.8, and 50×0.55 objectives, respectively. **b** SEM image of the composite glass-crystal pattern. The dotted lines are meant to visualize the inclinations of different textures. **c** The period as a function of the incident angle. **d** SEM images of the textures written with different light fluence (denoted by pulse energy).

Another important property is that the textures cannot be erased by the rewriting process. This is not surprising, since the inverse crystal-to-glass process is much more difficult than crystallization by laser direct writing. Thus, the textures with different inclinations can be in situ superposed together by the rewriting process, resulting in more complicated interlaced crystalline patterns in the glass. According to the theoretical model, the angle of the incident light actually participated in the interference relative to the optical axis is determined by the NA of the optical focusing system. Therefore, changing NA will change the direction of the incident light. This will lead to a change in the interference field and the inclination degree of the textures can be tuned. Here, we initially demonstrated that the tilt of the texture can be adjusted by changing the NA of optics. As shown in Fig. S8a and b, we successfully superpose three different textures together to form an unparalleled composite glass-crystal pattern by using different objectives. Such structures are once not achievable by any established technologies, but now can be finished within a few seconds, which offers an intriguing strategy to create complex inorganic structures. Meanwhile, except for the influence of optics, the physical environment at the focal area after tight-focusing and non-tight focusing generated by the optics with different NA could also be different. As a result, more other factors may also affect the interference field, including temperature, pressure, ionization, melting, crystal nucleation, and growth et al.

According to the theoretical model, the period of the texture could be adjusted by controlling several processing parameters (Equation S2-5), such as incident angle (θ) and light wavelength (λ). For instance, the period will decrease gradually with an increase in θ and the degree of this reduction is different at different depths (shown in Fig. S8c). The calculated largest difference in the period of the ripples with the angle of incidence in the range 0-90 deg is about 120 nm at the depth of 20 µm (the maximum size of the texture in the Z-axis direction). And at the depth of 5 µm and 15 µm, the period of the ripples hardly varies with incident angle (Fig. S8c). However, although the incident angle, in theory, can be changed from 0 to 90 deg, the achievable change in the incident angle is limited by the NA of optics, and it is unfeasible to adjust it in such a large range. Furthermore, due to the complicated ultrafast laser-matter interaction process, there are synergistic effects when applying a change to these parameters. For example, due to the optical dispersion effect, the dielectric media generally have different optical responses for light with various wavelengths, such as RI and non-linear absorption rate. Thus, applying a change to wavelength will lead to a shift in the focus state. We found that when using a 515 nm-laser as the light source, the LTN glass sample can hardly be processed. This is because the shortwave beam is difficult to be tightly focused in the high-refractive-index media. As a result, until now, it is difficult to induce an obvious change in the period by simply adjusting the NA or laser wavelength.

In addition, other laser parameters like pulse energy can also have an effect on the period of the textures. This effect is reflected in the degree of crystallization of the glass matrix. As higher laser fluence will lead to a stronger thermal effect in the interference field, the spacing of the ripples may shrink or even disappear, which is firmly confirmed by our experiments (Fig. S8d).

### S7 Demonstration of the universality of the SOPTL principle


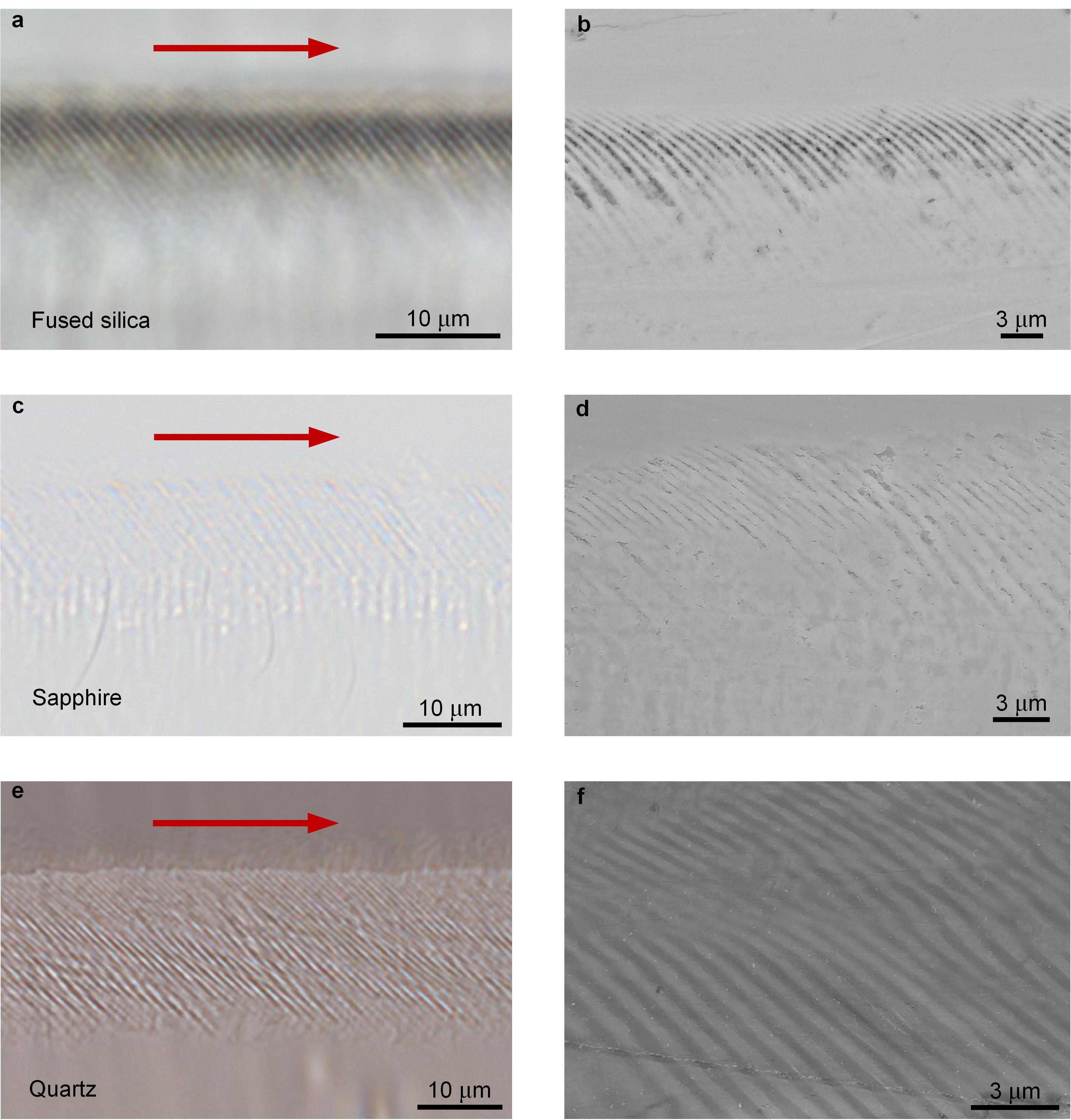


**Fig. S9 Periodic structures generated in other materials.** Optical and SEM images of the textures induced in **a, b** fused silica, **c, d** sapphire, and **e, f** quartz. The red arrows indicate the scanning direction.

To demonstrate the universality of the proposed physical model, we have applied it to more commonly used transparent dielectrics, including conventional glass (fused silica), and crystal (quartz and sapphire). As expected, similar tilted textures are formed in the beam writing area, which is proved by both optical microscopic observation and SEM observation (Fig. S9). All the induced textures tilt towards the starting point of laser scanning, which is in line with the theoretical prediction. These results demonstrate that the proposed physical model is applicable to a wide range of transparent dielectrics (not only in glasses but also in crystals). Notably, the inclination and bending degree of the textures induced in different materials may be different from one to another. This is because that the EPSs that participate in the formation of periodic structures will be different when processing in different transparent materials. More comprehensive and in-depth studies are needed in the future to fully understand this phenomenon.

### Supplementary References

1 Mainfray, G. & Manus, G. Multiphoton ionization of atoms. *Reports on Progress in Physics* **54**, 1333-1372 (1991).

2 Rajeev, P. P. *et al.* Memory in nonlinear ionization of transparent solids. *Physical Review Letters* **97**, 253001 (2006).

3 Mokkelbost, T. *et al.* Thermal and mechanical properties of LaNbO_4_-based ceramics. *Ceramics International* **35**, 2877-2883 (2009).

4 Huse, M. *et al.* Neutron diffraction study of the monoclinic to tetragonal structural transition in LaNbO_4_ and its relation to proton mobility. *Journal of Solid State Chemistry* **187**, 27-34 (2012).

5 Yashima, M., Lee, J. H., Kakihana, M. & Yoshimura, M. Raman spectral characterization of existing phases in the Y_2_O_3_-Nb_2_O_5_ system. *Journal of Physics and Chemistry of Solids* **58**, 1593-1597 (1997).

6 Feng, J., Shian, S., Xiao, B. & Clarke, D. R. First-principles calculations of the high-temperature phase transformation in yttrium tantalate. *Physical Review B* **90**, 094102 (2014).

7 Devan, R. S., Ho, W. D., Wu, S. Y. & Ma, Y. R. Low-temperature phase transformation and phonon confinement in one-dimensional Ta_2_O_5_ nanorods. *Journal of Applied Crystallography* **43**, 498-503 (2010).

8 Joseph, C., Bourson, P. & Fontana, M. D. Amorphous to crystalline transformation in Ta_2_O_5_ studied by Raman spectroscopy. *Journal of Raman Spectroscopy* **43**, 1146-1150 (2012).

9 Ma, X., Peng, Z., Li, J. & Mauro, J. Effect of Ta_2_O_5_ substituting on thermal and optical properties of high refractive index La_2_O_3_-Nb_2_O_5_ glass system prepared by aerodynamic levitation method. *Journal of the American Ceramic Society* **98**, 770-773 (2015).

10 Masuno, A. & Inoue, H. High refractive index of 0.30La_2_O_3_-0.70Nb_2_O_5_ glass prepared by containerless processing. *Applied Physics Express* **3**, 102601 (2010).
